# Retrospective study on the occurrence of *Salmonella* serotypes in veterinary specimens of Atlantic Canada (2012–2021)

**DOI:** 10.1002/vms3.1530

**Published:** 2024-07-09

**Authors:** Shivani Ojha, Krishna K. Thakur, Rasaq A. Ojasanya, Matthew E. Saab

**Affiliations:** ^1^ Department of Pathology and Microbiology Atlantic Veterinary College, University of Prince Edward Island Charlottetown Prince Edward Island Canada; ^2^ Veterinary Diagnostic Services Laboratory Atlantic Veterinary College, University of Prince Edward Island Charlottetown Prince Edward Island Canada; ^3^ Department of Health Management Atlantic Veterinary College, University of Prince Edward Island Charlottetown Prince Edward Island Canada

**Keywords:** antibiotic resistance, Atlantic Canada, exotic animals, laboratory data, *Salmonella*

## Abstract

**Aim:**

This study aimed to summarize the frequency and the antimicrobial susceptibility profiles of the *Salmonella* serotypes identified from the specimens of companion animals, livestock, avian, wildlife and exotic species within Atlantic Canada.

**Materials and Methods:**

The retrospective electronic laboratory data of microbiological analyses of a selected subset of samples from 03 January 2012 to 29 December 2021 submitted from various animal species were retrieved. The frequency of *Salmonella* serotypes identified, and their antimicrobial susceptibility results obtained using the disk diffusion or broth method were analysed. The test results were interpreted according to the Clinical and Laboratory Standards Institute standard. The *Salmonella* serotypes were identified by slide agglutination (Kauffman–White‐Le‐Minor Scheme) and/or the Whole Genome Sequencing for the *Salmonella* in silico Serovar Typing Resource–based identification.

**Results:**

Of the cases included in this study, 4.6% (*n* = 154) had at least one *Salmonella* isolate, corresponding to 55 different serovars. *Salmonella* isolation was highest from exotic animal species (*n* = 40, 1.20%), followed by porcine (*n* = 26, 0.78%), and canine (*n* = 23, 0.69%). *Salmonella* subsp. *enterica* serovar Typhimurium was predominant among exotic mammals, porcine and caprine samples, whereas *S*. Enteritidis was mostly identified in bovine and canine samples. *S*. Typhimurium of porcine origin was frequently resistant (>70.0%) to ampicillin. In contrast, *S*. Typhimurium isolates from porcine and caprine samples were susceptible (>70.0%) to florfenicol. *S*. Oranienburg from equine samples was susceptible to chloramphenicol, but frequently resistant (>90.0%) to azithromycin. In avian samples, *S*. Copenhagen was susceptible (>90.0%) to florfenicol, whereas Muenchen was frequently resistant (>90.0%) to florfenicol. *S*. subsp. *diarizonae* serovar IIIb:61:k:1,5 of ovine origin was resistant (50.0% isolates) to sulfadimethoxine. No significant changes were observed in the antibiotic resistance profiles across the study years.

**Conclusions:**

This report provides data for surveillance studies, distribution of *Salmonella* serotypes and their antimicrobial resistance among veterinary specimens of Atlantic Canada.

## Graphical abstract

This study highlights the carriage of uncommon *Salmonella* serovars among equine and wildlife animal species. Several serovars of *Salmonella* spp. displayed invasiveness. The wildlife serovars were susceptible to the commonly used antibiotics. The serovars Dublin from farmed mink and Typhimurium from porcine species were resistant to several antibiotics. It is impactful in the purview of public health that clinicians are encouraged to submit diagnostic samples and that the diagnostic lab continues to serotype salmonellae and test antimicrobials to support health initiatives.

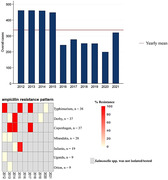

## 1 INTRODUCTION

*Salmonella* is a gram‐negative facultative anaerobic bacterium that causes enteric and invasive illnesses in both humans and animals. The geographical distribution of *Salmonella* is worldwide. It is carried in the intestinal tract of several animal species, including both homeotherm (warm‐blooded) and poikilotherm (cold‐blooded) animals that become reservoirs of its various serotypes (Grimont & Weill, 2007). The organism is often implicated in foodborne zoonoses, with serious public health concerns. Certain serotypes and strains can cause severe clinical conditions in their hosts, including gastroenteritis, septicaemia and abortion (Dietz et al., 2006; Gal‐Mor et al., 2014; Lamas et al., 2018).

The genus *Salmonella* is classified into two species, *Salmonella enterica* and *Salmonella bongori*. *S. enterica* is composed of six subspecies, including *enterica* (I), *salamae* (II), *arizonae* (IIIa), *diarizonae* (IIIb), *houtenae* (IV) and *indica* (VI). In addition to the phylogeny‐based classification of subspecies, *Salmonella* is further speciated into serotypes according to the Kauffman–White‐Le Minor scheme based on three major antigenic determinants: somatic (O), capsular (K) and flagellar (H) (Grimont & Weill, 2007). *S. enterica* subsp. *enterica* is also classified as typhoid or nontyphoid *Salmonella* (NTS) on the basis of their ability to cause specific pathologies in humans. Typhoid serovars, such as *Salmonella* Typhi, Sendai and Paratyphi A, B and C, are adapted to colonize a narrow range of hosts, including humans and higher primates. In contrast, NTS are generalist serovars capable of infecting both humans and animals (Gal‐Mor et al., 2014). The serotypes of *S. enterica* subsp. *enterica* are most frequently reported in gastroenteritis cases of NTS (Lamas et al., 2018).

In Canada, *Salmonella* (NTS) has been ranked as the second most acquired domestic foodborne pathogen, accounting for 24% of hospitalizations (Thomas et al., 2015). Most of the *Salmonella*‐related infections involving public health are linked to the sources of plant and/or animal agriculture, whereas 9% of human salmonellosis is caused by direct contact with animals (Hoelzer et al., 2011; Thomas et al., 2015). The majority of salmonellosis cases cannot be associated with outbreaks and are classified as sporadic cases (Guillier et al., 2021). Being an intestinal resident, *Salmonella* naturally contaminates the environment and circulates among different animal species, both domestic and wild, in a geographical area. The diversity of possible reservoirs of infection and rising antimicrobial resistance (AMR) of *Salmonella* pose challenges for public health authorities to control and treat the infections (La Tela et al., 2021; Marin et al., 2021; Wang et al., 2019).

Surveillance efforts and statistical estimates often focus on the agriculture and contamination points along the food chain to assess the source and risk of human infection. The role of diverse reservoirs in the dissemination of *Salmonella* and the circulation of antibiotic‐resistant serovars in different species is largely overlooked. The diagnostics of *Salmonella* at the serovar resolution is based on phenotypic methods as well as molecular methods (Grimont & Weill, 2007; Yoshida et al., 2016). The tools for analysing foodborne zoonoses, such as PulseNet, can identify *Salmonella* disease clusters during an outbreak. On the same note, the increasing trend of exotic pet‐related *Salmonella* serotypes can be detected through laboratory‐based surveillance (Centers for Disease Control and Prevention (CDC), 2011; Gambino‐Shirley et al., 2016). Besides detecting the pathogen, public health surveillance programs, such as CIPARS (Canadian Integrated Program for Antimicrobial Resistance Surveillance) investigate the trends of AMR of important foodborne pathogens (CIPARS, 2019a,b).

The Atlantic Canada Ecozone harbours a wide variety of terrestrial, aquatic wildlife, livestock and exotic pets. A human outbreak of *Salmonella* Typhimurium infection in multiple jurisdictions across Canada during 2017–2019 was source tracked to pet snakes and rodents. This outbreak also included three Atlantic provinces (Atlantic News, 2019). Because laboratory data provide crucial information on the trends and attributes of a pathogenic agent, the outbreak linked to exotic pets in Atlantic Canada prompted us to examine the retrospective laboratory data for a better understanding of the occurrence and antimicrobial susceptibility profiles of *Salmonella* serovars from samples in our region. In this study, we present the retrospective analyses of laboratory metadata on *Salmonella* identification and characterization spread over a 10‐year period.

## 2 MATERIALS AND METHODS

### 2.1 Data source

Records of microbiological analyses of samples from the bovine, porcine, equine, canine, avian, ovine, feline, caprine, exotic reptile, exotic avian and exotic mammal from 03 January 2012 to 29 December 2021 were analysed in this study. These samples were submitted from veterinarians based in the Atlantic Canadian provinces of Prince Edward Island, Nova Scotia, New Brunswick and Newfoundland & Labrador. As the objective of this study was to describe the frequency of *Salmonella* serovars, only specimens from the gastrointestinal tract (as these specimens always include *Salmonella* enrichment and selective culture) and specimens from any other body site that cultured *Salmonella* were included in the analysis. Samples were extracted from the Laboratory Information Management System based on their laboratory test request codes or positive *Salmonella* culture results. The samples tested consisted of clinical cases and surveillance/research projects; however, this could not be determined based on the data provided due to laboratory privacy agreements.

The search criteria run provided the variables of date of sample submission, sample identification number (a unique case number assigned by the laboratory), animal species, breed, anatomic site of the sample, type of specimen (e.g. swab, tissue, fluid and culture plates), bacterial organisms isolated and antimicrobial susceptibility test (AST) results. Samples submitted for bacterial culture and identification that were assigned the same identification number by the laboratory were referred to as individual cases for analysis in this study.

### 2.2 Bacterial culture and *Salmonella* detection

Aerobic bacterial culture of diagnostic specimens, such as swabs, tissues, fluids or faeces, was performed following standard bacteriological techniques and laboratory standard operating protocol (Quinn et al., 1994). *Salmonella* was identified as a differential among the members of *Enterobacterales* in the samples of clinical cases, whereas microbiological procedures to enhance the recovery of *Salmonella* (enrichment) were used in the gastrointestinal, surveillance and research samples (Poppe et al., 2004). However, the laboratory was unable to provide further categorization based on confidentiality agreements. Clinical history, including primary differentials, was generally not provided to the lab. Laboratory staff are trained to look for *Salmonella* in all clinical cases of gastrointestinal disease.

Between the years 2012 and 2020, all culture media were prepared by an in‐house media preparation laboratory, whereas from 2021 onwards, all culture media plates except Buffered Peptone Water (BPW) and Modified Semi‐Solid Rappaport Vassiliadis (MSRV) were purchased through Thermo Fisher Scientific Microbiology (Oxoid). BPW and MSRV continued to be prepared by an in‐house media preparation laboratory.

Specimens originating from an anatomic site other than gastrointestinal tract (except the oropharynx) were inoculated onto various culture media that included Columbia agar with 5% defibrinated sheep blood (BA) and MacConkey agar (MAC). Specimens from the gastrointestinal tract (excluding the oropharynx) were inoculated onto BA, MAC, Xylose Lysine Deoxycholate (XLD) or Xylose Lysine Tergitol 4 (XLT4) culture media. BA and MAC plates were incubated at 35°C with 5% carbon dioxide, whereas XLD and XLT4 plates were incubated at 35°C in ambient atmosphere. All agar plates were checked at 24 and 48 h. To selectively enrich for low numbers of salmonellae, gastrointestinal tract samples were also inoculated into either Rappaport Vassiliadis enrichment broth or BPW (both prepared by an in‐house media preparation laboratory). For screening specimens (e.g. checking for subclinical *Salmonella* spp. carriage) of the gastrointestinal tract, samples were inoculated directly into BPW enrichment broth. After incubation at 35°C, the enriched broths were plated onto MSRV agar for incubation at 42°C. The plates were checked daily for 3 days for typical swarming motility of *Salmonella* spp. (Poppe et al., 2004). Suspect *Salmonella* colonies were first recognized based on their colony morphology on the selective or non‐selective culture media.

Based on colony morphology on selective and non‐selective culture media, suspect *Salmonella* colonies were identified using matrix‐assisted laser desorption/ionization (MALDI) time‐of‐flight mass spectrometry and the Bruker MALDI Biotyper instrumentation and reference library (Bruker Daltonics Inc.). Each MALDI Biotyper analysis included the manufacturer's Bacterial Test Standard (extract of *Escherichia coli* DH5α), which was used to calibrate the instrument to eight reference peaks. Isolates with score values ≥2.00 were considered high‐confidence identification and were confirmed by serotyping.

### 2.3 Serotyping

At least one presumptive *Salmonella* isolate per case was submitted for serotyping. If multiple colony morphotypes were present in a single case, each morphotype would be submitted. For cases with multiple animals submitted, one isolate per animal was selected and submitted for serotyping to the Guelph Reference Services Unit at the National Microbiology Laboratory in Guelph and the World Organization for Animal Health (WOAH) Reference Laboratory for Salmonellosis. The isolates were serotyped using the Kauffman–White‐Le Minor scheme (June 2012–2020) or the Whole Genome Sequencing (WGS) for *Salmonella* in silico Serovar Typing Resource–based serovar identification (June 2020 to December 2021).

### 2.4 Antimicrobial susceptibility testing

AST was performed using either broth microdilution (for samples received after 1 August 2015) or the disk diffusion method following the Clinical and Laboratory Standards Institute (CLSI) testing methodology and interpretative criteria (M100 or VET01S) that were current at that time for bacteria isolated from animals or humans, as required (CLSI, 2013, 2015).

Disk diffusion testing was performed using panels that were designed in‐house and read manually using a ruler or vernier callipers. Disks were purchased from a commercial supplier (Thermo Fisher Scientific Microbiology [Oxoid]), and Mueller–Hinton agar was either prepared in‐house (2012–2020) or by a commercial supplier (2020–2021, Thermo Fisher Scientific Microbiology [Oxoid]). Broth microdilution was performed using the Sensititre Complete Automated AST System (Thermo Scientific) and commercial microdilution panels: Companion animals (dogs, cats, exotics) were tested using COMPAN1F, COMPAN2F or COMPGN1F panels; livestock (food/fur‐producing animals) were tested using BOPO6F or BOPO7F panels; horses and some exotic species (e.g. lagomorphs, guinea pigs) were tested using EQUIN1F or EQUIN2F panels (Thermo Fisher Scientific Microbiology, Oxoid). Quality control strains of *E. coli* ATCC 25922, *Pseudomonas aeruginosa* ATCC 27853, *Staphylococcus aureus* ATCC 29213 and *Enterococcus faecalis* ATCC 29212 were included within specifications of panels.

Antimicrobial susceptibilities to ampicillin, amoxicillin‐clavulanate, ceftiofur, cefovecin, cefpodoxime, chloramphenicol, doxycycline, enrofloxacin, florfenicol, marbofloxacin, oxytetracycline, sulphadimethoxine and trimethoprim‐sulfamethoxazole were included in the analyses. Antimicrobial susceptibilities were interpreted and categorized into two categories: susceptible and not susceptible (NS). The NS category combines the intermediate (moderately susceptible) and resistant results for the evaluated antimicrobials.

### 2.5 Data management

Retrieved data were checked for consistency and edited as necessary to group entries spelled differently or those missing identifications. Duplicate entries and bacteria tested for quality assurance purposes were excluded from the analysis upon cleaning and verification. Clearly identified anatomic sites, such as faeces, intestine, cecum, lung, liver, gallbladder and kidney, were maintained separately. Anatomic sites with missing records were grouped as ‘unknown’, whereas blood, bone, brain and so on were grouped as ‘others’. Environmental and unknown specimen sources from non‐animal species were excluded from the analysis.

In this study, ethical approval was not required as this was a secondary data analyses, and we have reported non‐identifiable data.

### 2.6 Statistical analysis

To summarize the metadata of common serotypes of *S. enterica* and their AST results, descriptive statistics were used. To classify the resistance trends of each *S. enterica* serotype, a logistic regression model was used with binary outcomes (susceptible and NS), to which a random effect was added to explain the clustering/correlation within cases from which the isolates were obtained.

For the data that met the inclusion criteria, the frequency of the samples submitted from animal species, average samples submitted per case, total cases submitted, *S. enterica* cases detected and *S. enterica* serotypes identified were summarized using Stata version 15 (StataCorp). *Salmonella* serotypes recovered from samples were presented as percentages with 95% confidence intervals calculated using the exact method. The annual and monthly frequency of *Salmonella* cases was graphically represented using bar charts. The frequency of *Salmonella* serotypes detected in various breeds of animal species was tabulated.

Antimicrobial susceptibility of *Salmonella* serotypes was reported for each animal species as percentages of isolates recorded as susceptible (S) and NS. Heat maps were used to visualize the annual AMR patterns for the most frequently tested *Salmonella* serotypes in 2 or more years using the pheatmap package (https://cran.r‐project.org/web/packages/pheatmap/index.html) (Kolde, 2018) in R Statistical Software (version 1.4.17; R Core Team, 2019).

Logistic regression models were used to assess the trends in AMR across the study years. For each *Salmonella* serotype tested for antimicrobial susceptibility, NS outcome (*R* = 1) was compared with the susceptible outcome (*R* = 0). The probability of AMR (*R* = 1) was modelled as the binary outcome with the study year as a continuous explanatory variable. The assumption of linearity, however, could not be met, and study years were finally modelled as categorical variables to assess inter‐annual differences in resistance patterns. The clustering of samples within the submitted cases was accounted for using a mixed effects logistic regression with cases as the random effect. Hosmer–Lemeshow goodness‐of‐fit assessed the model fit, and the Wald test was used to infer significant differences (*p *< 0.05) in resistance patterns.

## 3 RESULTS

**FIGURE 1:**
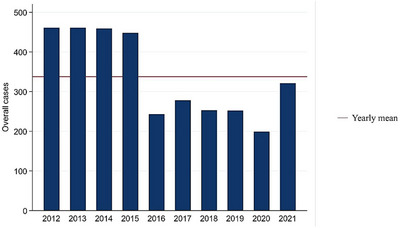
Annual frequency of cases submitted for bacterial culture and identification that met the inclusion criteria (2012–2021). The frequency of case submission was higher during the years 2012–2015, which dropped to less than half in 2020, suggesting SARS‐CoV‐2 restrictions.

**FIGURE 2:**
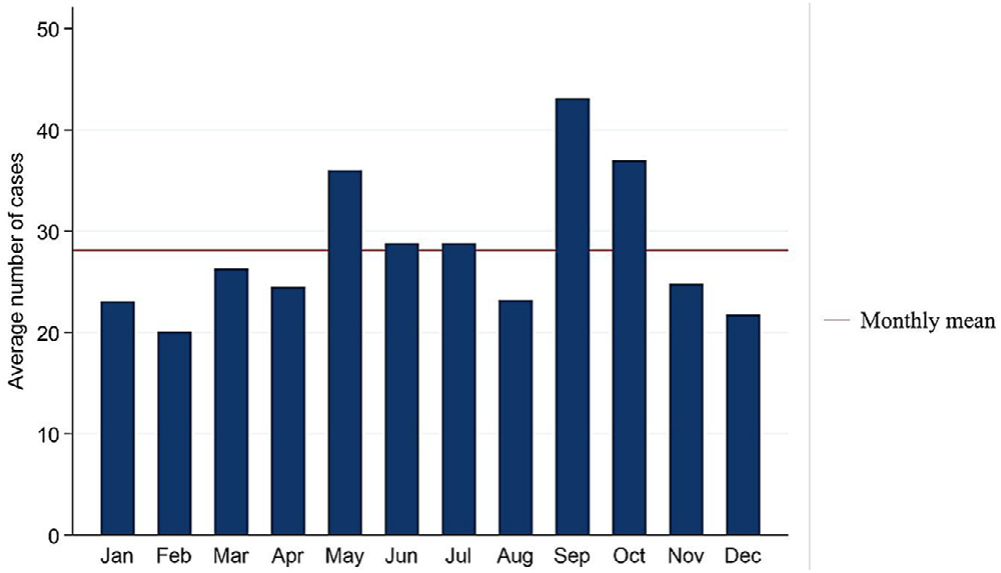
Monthly frequency of cases for bacterial culture and identification that met the inclusion criteria (2012–2021). The highest number of case submissions (>40) were recorded in the month of September, dropping to half in December and February.

The total number of samples submitted for bacterial culture that met the inclusion criteria of the study period was 11,792. The total samples submitted within the study period corresponded to 3,327 unique cases (defined as a group of samples submitted by clinicians on any given day for bacterial culture and identification) that met the inclusion criteria. Each case included a sample submission that ranged from 1 to 143 (mean = 3.54, median = 1.0) samples from a unique farm or owner on any given day. The average number of unique cases investigated that met the inclusion criteria per year and per month was 334 and 28, respectively. The annual and monthly frequencies of submitted cases that met the inclusion criteria are presented in Figures 1 and 2, respectively. The metadata of the same is included in Table S6A. On average, the laboratory received the highest frequency of cases in the month of September. The frequency of samples submitted from various animal species and their breeds or common names are summarized in Table S1.

The frequency of samples submitted, average sample submitted per case and cases with *S. enterica* serotype identification from samples in each animal species are summarized in Table 1. The highest frequency of cases submitted that met the inclusion criteria were from equine species (*n* = 1609, 48.3%), followed by bovine (*n* = 686, 20.6%), and canine (*n* = 322, 9.7%). Of the total cases submitted that met the inclusion criteria, 4.66% (*n* = 154) had at least one *Salmonella* isolate. *Salmonella* isolation was highest from reptiles (*n* = 40, 1.20%), followed by porcines (*n* = 26, 0.78%), and canines (*n* = 23, 0.69%). *Salmonella* ser. Typhimurium was highly represented among exotic mammals, porcine, and caprine samples. *Salmonella* ser. Enteritidis was mostly prevalent in bovine and canine samples, whereas in avian, ovine, equine and feline samples, *Salmonella* ser. Muenchen, *Salmonella* ser. IIIb:61:k:1,5, *Salmonella* ser. Anatum, *Salmonella* ser. IIIb:rough‐O:z:10, respectively, were identified. The frequency of anatomic sites and specimen types submitted during the study period that met the inclusion criteria is summarized in Table S2. Approximately 39.5% of the total samples submitted that met the inclusion criteria within the study period were faeces (*n* = 4660), and most submitted specimens were tissues (44.5%).

**TABLE 1:** Frequency of *Salmonella* cases and serotype identified from total samples submitted that met the inclusion criteria (2012–2021).

Animal species/source	The total frequency of samples submitted (*N*)	Average sample submitted per case (*N*)	Total case submitted (*N*)	Frequency of *Salmonella* case	Frequency of *Salmonella* serotype detection *n* (%)	*Salmonella* serotype 95% CI
**Bovine**	2285	3.33	686	7	Enteritidis 17 (0.74)	0.72–0.76
					Mbandaka 16 (0.70)	0.68–0.72
					Heidelberg 8 (0.35)	0.33–0.37
					*S. enterica* subsp. *diarizonae* 1 (0.04)	0.03–0.05
					Untypable 30 (1.31)	0.89–1.87
**Total detection**					**72 (3.15)**	
**Ovine**	864	7.02	123 (3.7)	12	IIIb:61:k:1,5 76 (8.80)	7.00–10.9
					Typhimurium 2 (0.23)	0.20–0.26
					IV:61:‐:1,5 1 (0.12)	0.10–0.14
					Untypable 2 (0.23)	0.20–0.26
**Total detection**					**81 (9.38)**	
**Caprine**	193	6.23	31	1	Typhimurium 16 (8.29)	4.41–13.11
**Total detection**					**16 (8.29)**	
**Porcine**	2097	21.0	100 (3.0)	26	Typhimurium 79 (3.77) 1:4, [5], 12:i: 17 (0.81) (monophasic)	2.99–4.67 0.79–0.83
					Derby 75 (3.58)	2.82–4.46
					Typhimurium var. Copenhagen 57 (2.72)	2.07–3.51
					Infantis 37 (1.76)	1.25–2.42
					Mbandaka 29 (1.38)	0.93–1.98
					Uganda 9 (0.43)	0.41–0.45
					Orion 9 (0.43)	0.41–0.45
					Untypable 80 (3.81)	3.04–4.72
**Total detection**					**392 (18.69)**	
**Equine**	1894	1.17	1609 (48.3)	10	Anatum 21 (1.11)	0.69–1.69
					Typhimurium var. Copenhagen 20 (1.05)	0.65–1.63
					Oranienburg 16 (0.84)	0.48–1.37
					Infantis 9 (0.48)	0.46–0.50
					Kentucky 8 (0.42)	0.40–0.44
					Senftenberg 1 (0.05)	0.04–0.06
					Barranquilla 1 (0.05)	0.04–0.06
					Untypable 38 (2.01)	1.42–2.74
**Total detection**					**114 (6.02)**	
**Canine**	1283	3.98	322	23	Enteritidis 38 (2.96)	2.10–4.04
					Heidelberg 32 (2.49)	1.71–3.50
					Panama 22 (1.71)	1.08–2.58
					I:–:d,i (rough) 19 (1.48)	0.89–2.30
					Reading 19 (1.48)	0.89–2.30
					Tennessee 19 (1.48)	0.89–2.30
					Hadar 19 (1.48)	0.89–2.30
					I:4,5,12:i 17 (1.33)	0.77–2.11
					Typhimurium 16 (1.24)	0.71–2.01
					Typhimurium var. Copenhagen 16 (1.24)	0.71–2.01
					I:roughO 7 (0.55)	0.04–0.07
					Heidelberg 7 (0.55)	0.04–0.07
					*S. enterica* subsp. *diarizonae* 1 (0.08)	0.06–0.10
					Newport 1 (0.08)	0.06–0.10
					Untypable 75 (5.85)	4.62–7.27
**Total detection**					**308 (24.01)**	
**Feline**	525	8.20	64	2	IIIb:Rough:z10:e,n,x,z15 19 (3.62)	2.19–5.59
					Heidelberg 16 (3.05)	1.75–4.90
					Untypable 1 (0.19)	0.00–1.06
**Total detection**					**36 (6.86)**	
**Avian**	970	6.18	157	14	Muenchen 24 (2.47)	1.59–3.66
					Typhimurium var. Copenhagen 16 (1.65)	0.95–2.67
					Bredeney 9 (0.93)	0.91–0.95
					I:4,[5],12:i‐3 (0.31)	0.28–0.34
					Typhimurium 1 (0.10)	0.08–0.12
					Senftenberg 1 (0.10)	0.08–0.12
					Untypable 12 (1.24)	0.64–2.15
**Total detection**					**66 (6.80)**	
**Exotic mammals**	1033	7.94	130 (3.9)	20	Typhimurium 57 (5.5)	4.20–7.09
					Heidelberg 51 (4.9)	3.70–6.44
					Kentucky 17 (1.6)	1.00–2.62
					Berta 16 (1.5)	0.81–2.38
					Dublin 16 (1.5)	0.81–2.38
					I:4,[5],12:i:−1 (0.44)	0.20–0.77
					I:4,12:‐2 (0.2)	0.02–0.70
					Hadar 1 (0.1)	0.00–0.54
					Typhimurium var. Copenhagen 1 (0.1)	0.00–0.54
					Untypable 53 (5.1)	3.87–6.66
**Total detection**					**215 (20.8)**	
**Exotic reptiles**	389	5.47	71 (2.1)	40	Kisarawe 32 (8.22)	5.69–11.41
					Fluntern 22 (5.66)	3.57–8.43
					Muenster 22 (5.66)	3.57–8.43
					II:16:m,t:−22 (5.66)	3.57–8.43
					IV:16 19 (4.88)	2.96–7.52
					IV:44: z4 19 (4.88)	2.96–7.52
					Blijdorp 16 (4.11)	2.37–6.59
					IIIb:48:z4,z24:−16 (4.11)	2.37–6.59
					Mountpleasant 16 (4.11)	2.37–6.59
					Paratyphi 5 (1.29)	0.42–2.97
					Hadar 4 (1.03)	0.28–2.61
					Blukwa 2 (0.51)	0.06–1.84
					I:4,5[12]:i:‐1 (0.26)	0.01–1.42
					II:58:I,z13,z28:z6 1 (0.26)	0.01–1.42
					IIIb:48:k:e,n,x,z15 1 (0.26)	0.01–1.42
					IIIb:53:z10:z35 1 (0.26)	0.01–1.42
					IIIb:60:i:e,n,x,z15 1 (0.26)	0.01–1.42
					IIIb:rough:z10 1 (0.26)	0.01–1.42
					IV:rough:g,z10 1 (0.26)	0.01‐1.42
					IIIa:18:‐1 (0.26)	0.01–1.42
					IV:50:g,z51:‐1 (0.26)	0.01–1.42
					Miami 1 (0.26)	0.01–1.42
					Poona 1 (0.26)	0.01–1.42
					Bareilly 1 (0.26)	0.01–1.42
					Benin 1 (0.26)	0.01–1.42
					Cerro 1 (0.26)	0.01–1.42
					Cotham 1 (0.26)	0.01–1.42
					Muenchen 1 (0.26)	0.01–1.42
					Rosslyn 1 (0.26)	0.01–1.42
					Untypable 7 (1.80)	0.73–3.67
**Total detection**					**219 (56.3)**	

The frequency of *S. enterica* serotypes isolated from samples of various animal species is summarized in Table S3. In exotic mammals, *Salmonella* ser. Typhimurium was the predominant serovar identified from mice, hedgehogs and ferrets, whereas *Salmonella* ser. Heidelberg was isolated from porcupines, mink and lions. *Salmonella* serovars Kentucky, Dublin and Berta were mainly isolated from mink.

Co‐isolation of *S. enterica* serotypes was observed in cases of exotic mammals and an exotic reptile. *Salmonella* serovars Kentucky and Heidelberg were identified together from lung tissues of a case, whereas *Salmonella* ser. Dublin was isolated from lungs of farmed mink in a different case submitted in 2013. *Salmonella* serovars Hadar and Heidelberg were present together in a peritoneal swab of porcupine (submitted in 2015). Similarly, co‐isolation of *Salmonella* serovars Typhimurium and Mbandaka occurred in two porcine cases (submitted in 2012). Co‐isolation of *Salmonella* serovars Mbandaka and Orion was also observed in a faecal sample from a different porcine case (submitted in 2013). Co‐isolation with multiple serovars of *Salmonella* was not observed in other animal species.

Antimicrobials tested and their corresponding CLSI susceptibility breakpoints for *S. enterica* isolates from various samples of animal species are presented in Table S4. The frequencies of *S. enterica* serotypes subjected to AST in each animal species are summarized in Table 2 and Table S5. Overall, AST was only analysed for *S. enterica* isolates that appeared in two or more samples of unique cases across various animal species. The porcine isolates of *Salmonella* serovars Typhimurium, Derby and ser. Typhimurium var. Copenhagen were frequently resistant (>70.0%; *n* = 36.86; 27.01; 37, respectively) to ampicillin, whereas *Salmonella* ser. Typhimurium var. Copenhagen and monophasic *Salmonella* ser. Typhimurium 1,4,[5],12:I‐ were frequently resistant (>90.0%; *n* = 32.4, 14, respectively) to florfenicol. In equine isolates, *Salmonella* ser. Oranienburg was susceptible to chloramphenicol but frequently resistant (>90.0%; *n* = 14.4) to azithromycin.

**TABLE 2:** Antimicrobial susceptibility profiles (% S and NS) for tested *Salmonella enterica* serotypes (2012–2021).

			Ampicillin			Azithromycin			Chloramphenicol			Clarithromycin			Florfenicol			Tetracycline	
Animal species	Bacterial isolates	N	S	NS	N	S	NS	N	S	NS	N	S	NS	N	S	NS	N	S	NS
Porcine	Typhimurium	38	2.6	97.4	–	–	–	–	–	–	–	–	–	35	74.3	25.7	–	–	–
	Derby	37	27.0	73.0	–	–	–	–	–	–	–	–	–	52	100.0	0.0	–	–	–
	Typhimurium var. Copenhagen	37	0.0	100.0	–	–	–	–	–	–	–	–	–	36	0.0	100.0	–	–	–
	Mbandaka	28	100.0	0.0	–	–	–	–	–	–	–	–	–	–	–	–	–	–	–
	Infantis	19	52.6	47.4	–	–	–	–	–	–	–	–	–	18	100.0	0.0			
	Uganda	9	100.0	0.0	–	–	–	–	–	–	–	–	–	–	–	–	–	–	–
	Orion	9	100.0	0.0	–	–	–	–	–	–	–	–	–	9	100.0	0.0	–	–	–
	I:4,[5],12:I:‐	–	–	–	–	–	–	–	–	–	–	–	–	16	0.0	100.0	–	–	–
Equine	Oranienburg	–	–	–	16	0.0	100.0	16	100.0	0.0	16	0.0	100.0	–	–	–	–	–	–
Avian	Muenchen	–	–	–	–	–	–	–	–	–	–	–	–	17	5.9	94.1	–	–	–
	Typhimurium var. Copenhagen	–	–	–	–	–	–	–	–	–	–	–	–	16	100.0	0.0	–	–	–
Ovine	IIIb:61:k:1,5	–	–	–	–	–	–	–	–	–	–	–	–	34	100.0	0.0	16	100.0	0.0
Caprine	Typhimurium	–	–	–	–	–	–	–	–	–	–	–	–	16	100.0	0.0	–	–	–

Abbreviations: N, number of isolates tested; **S**%, Susceptible; **NS**%, Not susceptible.

**FIGURE 3:**
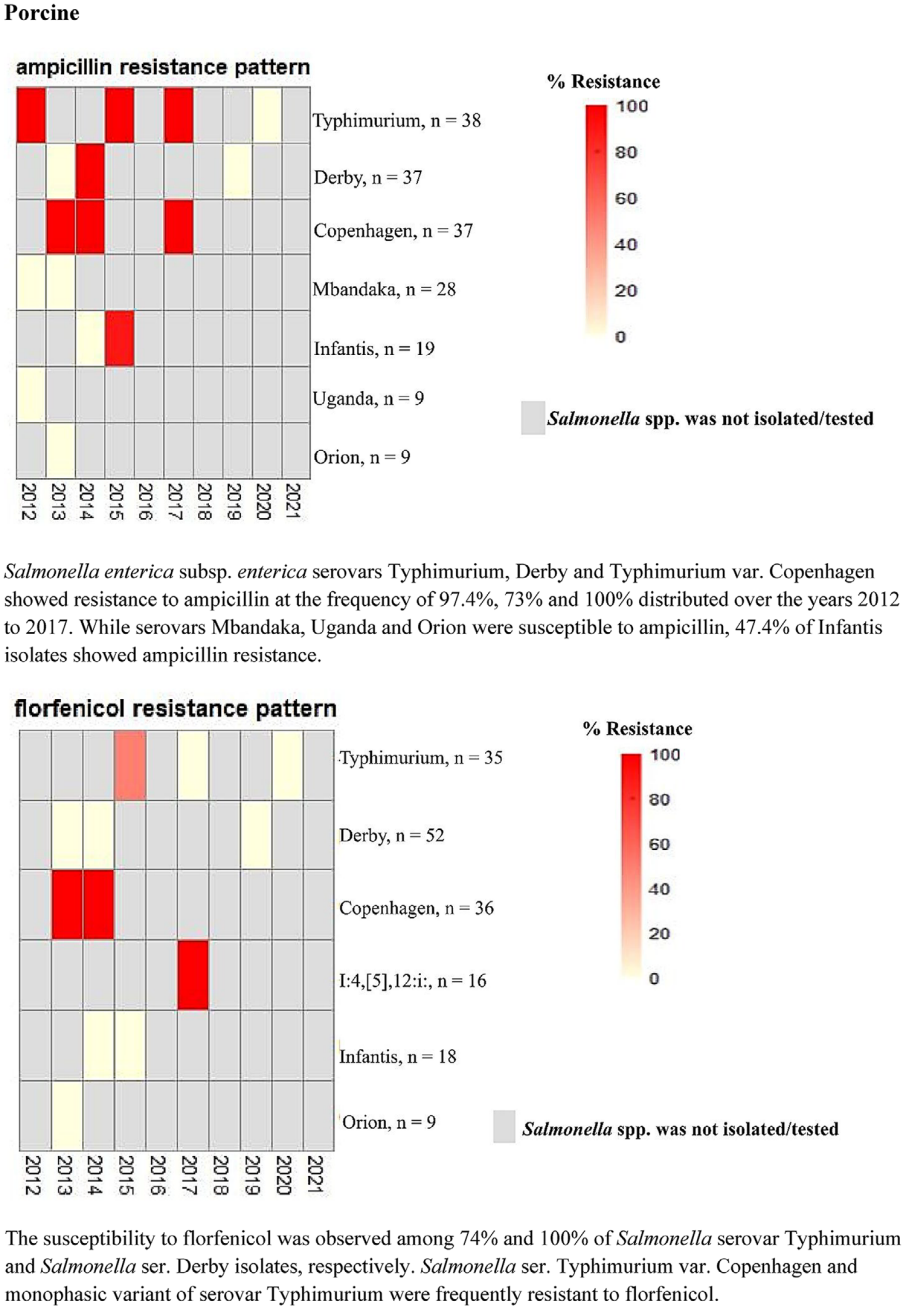
Heat maps showing annual antimicrobial resistance trends for the most frequently tested *Salmonella enterica* serotypes from porcine samples (2012–2021). The colour‐coding gradient represents % resistance of the *Salmonella* serovars to antibiotics. Red depicts the resistance of all the isolates of a serovar tested to an antibiotic (100% resistance), whereas a gradient of lighter shades to pale pink shows susceptibility. Grey bars represent either the absence of specific serovar of *Salmonella* or *Salmonella* isolates not subjected to antimicrobial susceptibility testing. *Salmonella enterica* subsp. *enterica* serovars Typhimurium, Derby and Typhimurium var. Copenhagen showed resistance to ampicillin at the frequency of 97.4%, 73% and 100% distributed over the years 2012–2017. While serovars Mbandaka, Uganda and Orion were susceptible to ampicillin, 47.4% of Infantis isolates showed ampicillin resistance. The susceptibility to florfenicol was observed among 74% and 100% of *Salmonella* serovar Typhimurium and *Salmonella* ser. Derby isolates, respectively. *Salmonella* ser. Typhimurium var. Copenhagen and the monophasic variant of serovar Typhimurium were frequently resistant to florfenicol.

The temporal AMR patterns for the most frequently tested *S. enterica* serovars in porcine species are presented with heat maps in Figure 3 (metadata in Table S6B). In caprine and equine, AST was predominantly conducted for *Salmonella* ser. Typhimurium and *Salmonella* ser. Oranienburg in 2017, respectively, so the heat maps were not generated. Overall, there were no significant inter‐annual differences in resistance patterns of *Salmonella* serotypes after accounting for the clustering of submitted samples within cases.

## 4 DISCUSSION

The analyses of laboratory data of a 10‐year period have provided comprehensive information on the trends of number of cases and sample submissions for *Salmonella* surveillance and detection, the prevalence of *Salmonella* serovars and their antimicrobial susceptibility profile. Although the sample submissions were higher from large animals, the total number of *Salmonella* detected, and thus serotyped, was highest from wildlife animal species. Moreover, a number of untypable isolates were less among the exotic reptiles compared to those from exotic and livestock animals. Besides faecal contents, *Salmonella* species were isolated from extraintestinal organs of wild and exotic animals, which suggests their invasiveness. In general, salmonellae from wildlife were susceptible to the tested antibiotics. It is noteworthy that *Salmonella* ser. Typhimurium isolated from porcine and wildlife samples was frequently resistant to ampicillin, sulphadimethoxine and oxytetracycline. It is interesting to note that although large numbers of submissions were received in the month of September, submissions decreased during the winter months of December, January and February, and in the summer month of August. During this decade, lowest sample submissions were made during the year 2020, which is suggestive of the restrictions because of the SARS‐CoV‐2 pandemic.

Although several of the serovars carried by the livestock animals are known and well documented, the exotic animal hosts, including but not limited to Reptilia, Amphibia, Mustelidae family, *Rodentia* and aquatic *Mammalia* remain the unknown shedders of many rare serovars of *Salmonella* in the environment. Many of such animals are either captive as pets, shop and educational exhibits, or live in the wild and directly or indirectly become the sources of *Salmonella* infections in humans (Abbott et al., 2012; Editorial Team et al., 2008; La Tela et al., 2021). In this text, the rarely known serovars identified from the equine species and the exotic species will be primarily discussed. The information on AST trends of the predominant *Salmonella* ser. Typhimurium, and the significance of wildlife in ‘One Health’ setting will be discussed.

There has been an ever‐increasing diversity of *Salmonella* serotypes, as these were first serotyped a century ago (Grimont & Weill, 2007). *S. enterica* subsp. *enterica* serotypes are principally related to livestock animals (warm‐blooded), and the other non‐*enterica* subspecies are generally found in the cold‐blooded animals (Lamas et al., 2018). However, this presumption is not exclusive, as both can be found in cold‐ and warm‐blooded animals; as was observed in our study, several *S. enterica* subsp. *enterica* serotypes were identified in reptiles and non‐*enterica Salmonella* subsp. IIIb serotypes were observed in sheep and, interestingly, in several specimens of cats. Despite various unique serovars identified, there are only a few serovars, such as *Salmonella* ser. Typhimurium and *Salmonella* ser. Enteritidis, which are usually associated with a clinical disease in humans (CDC, 2011; Tamber et al., 2021). These serovars were frequent in our dataset, of which *Salmonella* ser. Enteritidis was most common in bovine and canine species. The presence of *Salmonella* ser. Heidelberg in poultry is well known (Lefebvre et al., 2008). It is noteworthy that serovar Heidelberg was identified in samples of companion animals (32 in dogs; 16 in cats), porcupines and lions in the current study. The possibility of a raw chicken diet is hypothesized for the presence of *S*. Heidelberg in faeces of companion animals (Lefebvre et al., 2008).

*Salmonella* ser. Oranienburg isolates were identified from 16 samples of horse faeces in our laboratory. The large animal patients can potentially shed *Salmonella* in large numbers under stress. The nosocomial infection and environmental contamination associated with *Salmonella* ser. Oranienburg from equine index cases have been previously reported (Cummings et al., 2014; Jay‐Russell et al., 2014). Although the NTS serovar Oranienburg (O:7 group) is lesser known in animals, it has an epidemiological link in food poisoning outbreaks worldwide (CDC, 2007; Ooka et al., 2022; Werber et al., 2005), including a recent outbreak in the USA that was source tracked to fresh onion imports (CDC, 2021). The adaptation and persistence of *Salmonella* ser. Oranienburg could be attributed to its food‐related outbreaks (González‐Torres et al., 2023). Interestingly, *Salmonella* ser. Oranienburg was identified from paws and faecal samples of raccoons in Ontario in a surveillance study (Bondo et al., 2016). This serovar is one of the 19 NTS serovars that showed a higher rate of bacteraemia in human infections in Canada and Japan (Ooka et al., 2022; Tamber et al., 2021). Likewise, its invasiveness, leading to abscesses and haemorrhagic cystitis, has been reported (Castlemain & Castlemain 2022; Katsuno et al., 2003; Teh et al., 2017).

Atlantic Canada is home to a variety of terrestrial and aquatic free‐living wildlife species. The laboratory data of the past decade highlight the presence of some sparingly known serovars of *S*. subsp. *enterica* (I), whereas many specimens from wildlife show the presence of non‐*enterica* subspecies (II, III and IV). Recently isolated *Salmonella* species in 2020–2021 from several systemic and faecal specimens of porcupine and deer were untypable in the current study. The largest submission from the exotic mammals (ferrets, hedgehogs and mice) yielded *S. enterica* subsp. *enterica*, with *Salmonella* ser. Typhimurium being the predominant serovar, followed by *Salmonella* ser. Heidelberg from a set of faecal samples from lions and peritoneal cavity from porcupines. In a Health Canada survey, the *Salmonella* serovars Enteritidis, Typhimurium and Heidelberg were reported in large numbers, among which, serovar Heidelberg conspicuously showed a higher rate of bacteraemia (Tamber et al., 2021).

Interestingly, besides *Salmonella* serovars Heidelberg, Kentucky and Berta, a host‐adapted *Salmonella* ser. Dublin was detected in mink samples (*Neogale vison*) in 2013. Although Dublin is host adapted to cattle, the serovar has been reported in fur‐producing animals of mink and foxes in Denmark, causing reproductive losses (Dietz et al., 2006). This serovar is invasive and was isolated from the lungs, liver, intestines and lymph nodes specimens of the farmed mink. Besides the host's weak immune response, the high pathogenicity of *Salmonella* ser. Dublin is attributed to its virulence plasmids and its antibiotic‐resistant genes, leading to treatment failures (Harvey et al., 2017). *Salmonella* ser. Dublin from mink showed multi‐drug resistance (MDR) to azithromycin, penicillin, doxycycline and chloramphenicol.

Among the aquatic mammals, the harbour porpoise (*Phocoena phocoena*), which is distributed in the coastal waters of the Northern Atlantic and Pacific, has been identified as sentinels of the marine ecosystems (Sandholt et al., 2021). A monophasic group B *S. enterica* subsp. *enterica* with the antigenic formula 4,12:a:‐ is known in harbour porpoises in Scotland and England (Sandholt et al., 2021). The distribution of similar strains is not known in the Atlantic Canada waters. In our study, *S. enterica* of serogroup B with the antigenic formula 4,12:‐:‐ (non‐motile) was detected in the lung tissues of harbour porpoise and white‐beaked dolphin (*Lagenorhynchus albirostris*) from the Atlantic coast of Canada.

The global information on the carriage of *Salmonella* among the class of Reptilia is constantly increasing, and the diagnostic laboratory data from the Atlantic Canada region is an adjunct. Although a recent surveillance study in Europe isolated a lesser number of *S. enterica* subsp. *enterica* serovars from the cold‐blooded animals vs. warm‐blooded ones (La Tela et al., 2021), our dataset represented the serovars of four subspecies (I–IV) of *S. enterica* in reptiles, where sparingly known serovars were highly represented. Among these, the serovars Kisarawe, Cotham, Fluntern, Mountpleasant, Blijdorp and Blukwa were notable. Although *Salmonella* ser. Muenster of group E was identified in 5.6% of the isolated salmonellae from the oral cavity of lizards (year 2016), serovar Fluntern of O:18 group was isolated from oral cavity tissue submission of year 2018 from lizards. *S*. Muenster was reported from faecal and oral samples of captive reptiles in Spain (Marin et al., 2021). The serovar Muenster is associated with foodborne illnesses worldwide (Van Cauteren et al., 2009); however, it was not identified in any samples of avian or food animals in our study dataset. *Salmonella* ser. Fluntern has been implicated in multi‐state cluster illness in humans upon contact with geckos, a popular pet reptile (Koski et al., 2019), and can cause pyogenic infection in these animals (Zajac et al., 2020). Zajac et al. (2021), analysed more than 700 samples of different reptiles in Poland, which demonstrated the occurrence of Fluntern in lizards, snakes and their environment. In reptiles, *Salmonella* is spread by faecal–oral route. Isolation of *Salmonella* from the oral cavity heeds caution to the potential contamination of the reptile's skin, the environment and the sites of infection for reptile handlers.

*Salmonella* ser. Kisarawe of O:11 (F) serogroup is found in reptiles, and in the current study, it was identified from the faecal samples of bearded dragons in the year 2014. It has been isolated from bearded dragons in Europe as well (Lukac et al., 2015) and recently in a multi‐state outbreak in the USA of illness primarily in children under 5 years of age after contact with this reptile (Kiebler et al., 2020). The serovar Cotham appeared to be relatively invasive, as suggested by its isolation from the livers of bearded dragons in our study.

Another lesser reported *Salmonella* ser. Blijdorp was isolated from 16 faecal samples of chameleon (*Chamaeleo calyptratus*) in 2014, whereas a similar finding was reported in an active surveillance of captive veiled chameleons (Romero et al., 2015).

The global incidences of human infections associated with the Java variant of *Salmonella* ser. Paratyphi B has increased since 1990 and are mainly related to exposure to aquatic animals or reptiles (Hernández et al., 2012; Krishnasamy et al., 2018; Levings et al., 2006). The submission of snake livers in 2018 showed invasion by the d‐tartrate‐fermenting, non‐paratyphoidal *S. enterica* serovar Paratyphi B, var. Java.

Besides *S. enterica* subsp. *enterica* serovars, a decade of data has identified subspecies II, IIIa, IIIb and IV serovars from the members of the class Reptilia; but notably, none of these were identified from warm‐blooded exotic mammals. The serovars of subspecies IIIb and IV were found in extraintestinal tissues. The *S. enterica* subsp. *salamae* (II) O:16 serogroup was identified from colons of snakes (Ball python) as monophasic, devoid of phase 2 flagellar antigens but encoding m and t phase 1 antigens. The recombination events between m,t flagellin antigens among group II salmonellae have generated great diversity in flagellin serotypes, of which the m,t are the examples of complex mosaics owing to intragenic recombination events (Li et al., 1994).

The diagnostic data exhibited the presence of Arizona group *S. enterica* subsp. *arizonae* (IIIa) and *diarizonae* (IIIb) in exotic animals. Recently, *S. enterica* subsp. *arizonae* (IIIa) was reported in a case of canine prostatitis (Hertzer et al., 2021). In the current study, a non‐motile O:18 serovar of *S. enterica* subsp. *arizonae* (IIIa) was identified only from one faecal specimen of a snake, whereas *S. enterica* subsp. *diarizonae* (IIIb) was detected in several samples of reptiles and warm‐blooded animals. *S. enterica* subsp. *diarizonae* was largely detected from ovine faecal specimens (8.8%), and interestingly, rough variants of its serovar from cat faeces (3.6%). The serovars of *S. enterica* subsp. *diarizonae* were identified from liver samples of snakes and from samples of turtles in 2014. Although *S. enterica* group III subspecies are known to be harboured by poikilotherms, *S. enterica* subsp. *diarizonae* is frequently reported from the environment, sheep and humans (Lamas et al., 2018). The adaptation of *S. enterica* subsp. *diarizonae* in sheep is not well understood; however, the possession of type 6 secretion system (T6SS), gain of *Salmonella* Pathogenicity Island‐18 and specific fimbriae in the course of divergence could be the survival advantage factors for IIIb strains (Lamas et al., 2018). It is noteworthy that, in contrast to other non‐*enterica* subspecies, the Arizona group carries a T6SS similar to *Salmonella* ser. Typhimurium. It is thought that T6SS effectors provide niche‐occupying advantage to these subspecies in the gut by eliminating the local commensals (Desai et al., 2013).

*S. enterica* subsp. *houtenae* (IV) comprises less than 1% of all *Salmonella* strains (Grimont & Weill, 2007). It is known to be carried in wild animals as it was originally isolated from a cockatiel (*Nymphicus hollandicus*) in 1978, and 73 serotypes of *S. enterica* subsp. *houtenae* have since been described (Lamas et al., 2018). Although *S. enterica* subsp. *houtenae* is common in reptiles and amphibians (Mermin et al., 2004), it was recently reported from a neck abscess in Roe Deer (*Capreolus capreolus*) (Trotta et al., 2021). In our study, *S. enterica* subsp. *houtenae* serovar O:44 was identified in 19 surgical swab samples from bearded dragons. Such observation highlights the latent carriage of the organism on the skin of poikilotherms, which, if contacted by the homeotherms, can outgrow at higher temperatures to cause illness. The *S. enterica* subsp. *houtenae* serovars of O:44 group, a rough serovar (–:g,z10:‐) and mucoid phenotype of O:50 group were identified from systemic organs of reptiles in the current study. The non‐motile serovar exhibiting O:16 antigens (16:‐:‐) was found in the oviduct swabs of snakes in the year 2019. Although the serovar O:16 was reported in an HIV patient in Brazil (Lourenco et al., 2004), the *S. enterica* subsp. *houtenae* was noted as the most prevalent subsp. in human salmonellosis in Germany with known exposure to reptiles (Editorial Team et al., 2008). Several clinical case presentations are suggestive of an opportunist invasive behaviour of *S. enterica* subsp. *houtenae* serovars (Abbott et al., 2012; Editorial Team et al., 2008; Nimir et al., 2011; Tabarani et al., 2010).

In our data, the sample distribution of reptiles and exotic mammals is well represented over the 10‐year period. A Health Canada survey has reported the bacteraemia‐associated serovars, of which, the serovars Paratyphi, Heidelberg, Hadar, Muenchen, Poona, Bareilly, Muenster and Dublin were found in the exotic species of Atlantic Canada. Of note, the bacteraemia‐causing serovars are taxonomically *S. enterica* subsp. *enterica* strains, whereas non‐*enterica* serovars have not been reported in severe cases of infections. The absence of virulence genes is attributed to the limited pathogenicity of non‐*enterica* serovars, restricting their invasive potency (Lamas et al., 2018). However, the non‐*enterica* serovars are capable of causing severe illness in immunocompromised adults and children and have been detected in immunosuppressed companion animals as well; henceforth, these are argued as being the ‘opportunists’ (Hertzer et al., 2021; Krath et al., 2020; Lamas et al., 2018; Tabarani et al., 2010).

Although few recent outbreaks of salmonellosis in the Maritimes were traced to exotic pets, the contribution of wildlife in the dissemination of *Salmonella* and on the maintenance of antibiotic‐resistant serovars is not extensively known (Atlantic News, 2019; Atlantic Brief Desk, 2023). Wildlife, in general, is considered sentinels of infectious agents and a disseminator of AMR (La Tela et al., 2021; Torres et al., 2020). The treatment regimen of *Salmonella* infection in humans includes third generation cephalosporins, quinolones and azithromycin (World Health Organization [WHO], 2023). A 2019 CDC report on salmonellosis has indicated a 10% rise in resistance to ciprofloxacin in NTS human isolates. However, generally, *Salmonella* ser. Typhimurium of porcine origin in our study and the analyses of United States National Antimicrobial Resistance Monitoring System (NARMS) metadata showed relatively lower resistance to quinolones (Wang et al., 2019). The 2019 CIPARS report highlights resistance of the commonly isolated *Salmonella* ser. Enteritidis from humans and broiler chickens to nalidixic acid in different provinces, including Atlantic Canada region (CIPARS, 2019b); however, our data showed susceptibility of canine isolates of this serovar to quinolones but resistance to ampicillin. Macrolides are used in human patients, as well as in horses, to treat bacterial infections (Gomes et al., 2017). However, most *Salmonella* strains are known to carry macrolide‐resistant genes on plasmids and show an elevated minimum inhibitory concentration to these antimicrobials (Gomes et al., 2017; Jia et al., 2023). The resistance to azithromycin was noted in the *Salmonella* serovars of wildlife and farmed animal species, as well as in *Salmonella* ser. Oranienburg from horses in our study.

Several studies have pointed out the carriage of AMR pathogens, including salmonellae in wildlife, citing anthropogenic reasons (La Tela et al., 2021; Torres et al., 2020; Vittecoqu et al., 2023). Notably, our findings of *Salmonella* AST in wildlife mirror those of Bondo et al. (2016, 2019) and Vogt et al. (2021) surveillance studies in Ontario, which found reduced AMR in *Salmonella* strains from free‐living wildlife, as well as pan‐susceptible *Salmonella* serovars from raccoons and swine environment. These observations are highly suggestive of a lack of direct antimicrobial selection pressure on the enteric bacteria of wildlife species, as opposed to agricultural animals receiving generic antimicrobials. Contextually and interestingly, the existence of the natural antibiotic susceptibility was suggested in subsp. *enterica* and *arizonae* (Stock & Wiedemann, 2000); however, acquiring resistant genes under selection pressures can make a difference to their infection outcomes.

In extensive in silico analyses of globally isolated *Salmonella* genomes, the abundance of AMR genes in group I *Salmonella* serovars, Typhimurium and its monophasic variant I:4,[5],12:i:‐ followed by serovars of group IIIb was demonstrated (Jia et al., 2023). In our study, the ovine and exotic mammals’ serovars of group IIIb were mostly sensitive to the antibiotics tested, except for a frequency of 50% resistance to sulphadimethoxine in sheep‐adapted isolates of IIIb 61:k:1,5. This observation was similar to the findings of high resistance of serovar 61:k:1,5 to sulfamethoxazole (Bonke et al., 2012; Methner & Moog, 2018).

The globally distributed *Salmonella* ser. Typhimurium appeared as the most represented serovar in food animals (porcine and caprine) as well as in exotic mammals in our study. The CIPARS (2019a) highlighted a significant increase in resistance of *Salmonella* ser. Typhimurium of porcine origin to ampicillin between 2010 (27%) and 2019 (47%). Several reports emphasize the persistence and transmission of resistant strains of *Salmonella* ser. Typhimurium through the food chain (CDC, 2019, 2021; Wang et al., 2019) and by direct contact with animals and raw pet foods (Atlantic News, 2019; Atlantic Brief Desk, 2023; Public Health Agency of Canada [PHAC], 2023). It is interesting to note that the antibiotic susceptibility patterns of ASuT (ampicillin, sulphonamides and tetracycline) and ACSuT (ampicillin, chloramphenicol [prohibited in food animals], sulphonamides and tetracycline) showed similar patterns of resistance to ampicillin, sulphadimethoxine and oxytetracycline among the *Salmonella* ser. Typhimurium isolates of exotic mammals and porcine origin. This observation reminds of the importance of biosecurity in commercial swine operations, as free‐living animals could be the vectors of MDR salmonellae (Bondo et al., 2019; La Tela et al., 2021; Vogt et al., 2021). The patterns of ampicillin, sulphonamides and tetracycline resistance in *Salmonella* serovars isolated from different sources are corroborated worldwide (Nguyen et al., 2016; Vogt et al., 2021; La Tela et al., 2021; Jia et al., 2021), including analyses of NARMS metadata by Wang et al. (2019), which illustrated a high frequency of tetra‐resistant ASSuT and penta‐resistant patterns of ACSSuT (including streptomycin) in *Salmonella* ser. Typhimurium strains across the food chain. Such findings, irrespective of geographic locations, are highly suggestive of generic antibiotics usage, enabling the selective emergence of resistant clones. The NARMS data are intriguing, as the high diversity in antibiograms of human *Salmonella* ser. Typhimurium strains compared to those from animal sources highly indicates diverse sources of human isolates (Wang et al., 2019). Besides, the high frequency of AMR in *Salmonella* is displayed in surveys of Asian countries where policies on antimicrobial use in human and animal sectors are not as stringent as in developed countries (Jia et al., 2023; Mechesso et al., 2020; Ripon et al., 2023). It is important to note that the resistant porcine isolates in our study belonged to the dataset prior to 2018, after which the medically important antibiotics became prescription‐authorized (Canadian Food Inspection Agency [CFIA], 2018).

The porcine isolates of the monophasic *Salmonella* ser. Typhimurium (I:4,[5],12:I‐) in our study were frequently resistant to florfenicol drug, which is widely used in swine operations to treat respiratory infections. This emerging monophasic serovar in human infections and in wildlife is noteworthy (Elnekave, et al., 2018; Jia et al., 2023; La Tela et al., 2021; PHAC, 2023), including the outbreak clade of MDR isolates originating from pork or contact with swine (Elnekave, et al., 2018; Plumb et al., 2023). In a recent outbreak involving Atlantic Canada provinces, the source of MDR I:4,[5],12:I‐ was raw pet food and contact with dog and cattle that led to hospitalizations, and 43% of cases were children less than 5 years of age (PHAC, 2023).

Recent studies at the genomic level have presented several highlights, such as the carriage of key AMR genes of beta‐lactams (*bla*_TEM‐1_, *bla*_NDM_), tetracyclines (*tetA, tetB, tetX*), colistin (*mcr1.1*) and macrolides in *Salmonella* serovars; the role of plasmids, plasmid‐borne prophages, *Salmonella* Genomic Island 1 in multidrug resistance; the abundance of AMR genes among *Salmonella* strains of animals and human origin vs. those from environmental sources; and the likelihood of resistant gene transfer in wildlife residing proximal to the animal facilities (Jia et al., 2023; Vogt et al., 2021; Wang et al., 2019). These studies have noted animal products and contaminated environment with resistant bacteria and antibiotic spills as potential links of AMR gene conduction across species. Although the transmission of *Salmonella* to humans usually occurs via foods or direct contact with animals, notably, the consumption of exotic meat products is on the rise (Muehlenbein, 2016). The persistence of a MDR, ciprofloxacin‐resistant *Salmonella* ser. Kentucky ST198, in a veterinary hospital environment was recently reported to cause hospital‐associated infections 7 months after its original isolation and was sourced back to a hospitalized American black bear (Cummings et al., 2023). In addition, the role of ecology and anthropogenic habitats in sustaining and propagating AMR has been examined in several studies (González‐Torres et al., 2023; Torres et al., 2020; Vogt et al., 2021). With the inception of ‘One Health’ paradigm, the complex role of wildlife in the distribution and maintenance of resistant clones at the interfaces of food animals, food chain and ecology is increasingly being emphasized (Cummings et al., 2023; González‐Torres et al., 2023; La Tela et al., 2021; Torres et al., 2020; Vogt et al., 2021).

*Salmonella* isolates of our study were generally susceptible to different classes of antibiotics, which points at the probable reason of lower number and less intensive livestock operations in Atlantic Canada, thereby reducing environmental contamination as well. It will be interesting to know the microbiomes of livestock and wildlife of the region, as the exchange of AMR genes is highly dynamic among gut microbiota (Jia et al., 2023). In a multi‐province salmonellosis outbreak of early 2023, the source of *Salmonella* ser. Typhimurium infection was linked to snakes and rodents that caused illness in 45 individuals, including children under 5 years old (Atlantic Brief desk, 2023). This outbreak reported one fatality. The high proportion of children affected in various outbreaks highlights the need to educate owners about the risk of exotic pets associated salmonellosis in children, the household pets and the potential for household contamination by pet reptiles (Hernández et al., 2012; Krishnasamy et al., 2018; Kiebler et al., 2020; Krath et al., 2020).

The present study, being not a prospective but a retrospective study, has its own limitations: (i) several *Salmonella* isolates were untypable, and we were unable to further analyse these strains, (ii) not an optimum representation of Atlantic Canada since submission of food animal samples limited to certain geographic locales, (iii) sample processing bias in samples from systemic organs that were devoid of *Salmonella* enrichment step vs. faecal samples and *Salmonella* surveillance samples, (iv) missing information on clinical vs. surveillance samples, (v) unable to confirm interpretative criteria for AST when using the disk‐diffusion method and (vi) reliability solely on electronic recordkeeping.

In addition, a primary limitation of comparing antimicrobial susceptibility data across studies, regions or animal species because of the differences and/or unavailability of the interpretative criteria used. For example, CLSI uses clinical breakpoints specific for the animal host, bacterium and antimicrobial and most often these clinical breakpoints are much lower for veterinary species when compared to humans. Culture methodology across studies could also introduce bias when comparing the proportion of samples positive for *Salmonella*.

## 5 CONCLUSIONS

The laboratory data of the Atlantic Canada give an estimate of the proportion of different *Salmonella* serovars detected among the analysed specimens from various animals of the region. Detection of unique serovars in equine and wildlife species highlights the importance of continued surveillance to protect the health of humans, animals and the environment to align with the concept of ‘One Health’. Reviewing the data in the context of similar reports also prompts the importance of infection control precautions during animal movements between various sites, including animal hospitals.

Although there are inherent flaws of examining the retrospective data, this study has provided a scaffold for hypotheses to work on. Active targeted surveillance of both captive and free‐living wildlife animal population in tandem with the use of advanced molecular epidemiological methods, such as WGS is desirable to solidify the information on *Salmonella* distribution in the region and to dissect out the virulence attribution of *Salmonella* species. As a next step, examination of the gut microbiome of the wildlife and a dominant food animal species of the region will be useful to comprehend the information on the drivers of AMR gene amplification in the wild.

## AUTHOR CONTRIBUTIONS

**Shivani Ojha**: Conceptualized the study; supervision; writing review and editing. **Krishna K. Thakur**: Investigation; methodology; resources; supervision; investigation of the data and supervision on the analytical methodology. **Rasaq A. Ojasanya**: Data curation; formal analysis; validation; writing certain sections of original draft. **Matthew E. Saab**: Data curation; resources; software; validation; writing review and editing.

## CONFLICT OF INTEREST STATEMENT

The authors declare no conflicts of interest.

### ETHICS STATEMENT

None.

### PEER REVIEW

The peer review history for this article is available at https://publons.com/publon/10.1002/vms3.1530.

## Supporting information

Supporting Information

## Data Availability

The data that support the findings of this study are available from the corresponding author upon reasonable request.
