# Supplementary material for: Retrospective study on the occurrence of Salmonella serotypes in veterinary specimens of Atlantic Canada (2012–2021)
Source: Vet Med Sci. 2024 Jul 9;10(4):e1530. doi: 10.1002/vms3.1530 (PMC11231644; doi:10.1002/vms3.1530)
Supplement: Supplementary file 1 — Supporting Information [file VMS3-10-e1530-s001.docx]

**Supplementary Table 1:** Frequency of samples submitted that met the inclusion criteria from various animal species and their breeds or common names (2012 – 2021)

| **Animal species** | **Breed / Common name** | **Frequency of samples submitted**  **N (%)** |
| --- | --- | --- |
| Bovine | Holstein-Friesian, Black Angus, Simmental, Charolais, Red Angus, Ayrshire, Jersey, American Wagyu, Hereford, Limousin, Beef master, Shorthorn, Mixed | 2,285 (19.4) |
| Porcine | Berkshire, Mixed | 2,097 (17.8) |
| Equine | Standardbred, Quarter Horse, Warmblood, Thoroughbred, Hanoverian, Paint, Miniature Horse, Belgian, Clydesdale, Welsh, Appaloosa, Percheron, Mixed, Morgan, Arabian, Norwegian Fjord, Friesian, Newfoundland Pony, Shetland, West Phalian, Trakehner, Oldenburg, Andalusian, American Cream, French Canadian, Light Horse, American Saddle Horse, Pony Of The Americas, Shire, Anglo-Arab, Connemara, Hafflinger, Icelandic, Palomino, Paso-Fino | 1,894 (16.1) |
| Canine | Golden Retriever, Mixed, Staffordshire Terrier, Poodle, German Shepherd, Chihuahua, Great Dane, Labrador Retriever, Cocker Spaniel, Beagle Hound, Shetland Sheepdog, French Bulldog, Yorkshire Terrier, Brittany Spaniel, Newfoundland, Rottweiler, Pug, Shih Tzu, Norwegian Elkhound, Boxer, Bull Mastiff, Bernese Mountain Dog, Golden Doodle, Greyhound, Maltese, Nova Scotia Duck Tolling Retriever, Australian Shepherd, Pomeranian, Border Collie, Siberian Husky, Labradoodle, Mastiff, Miniature Schnauzer, Boston Terrier, Bulldog, Dachshund, Saint Bernard, Samoyed, Cavalier King Charles Spaniel, Dalmatian, Jack Russel Terrier, Papillon, Portuguese Waterdog, West Highland Terrier, Wheaten Terrier, Borzoi, Chinese Crested, Cock-A-Poo, Doberman Pinscher, German Pinscher, Bichon Frise, Havanese, Norwich Terrier, Rhodesian Ridgeback, Scottish Terrier, Toy Fox Terrier, Australian Cattle Dog, Coton De Tuler, English Pointer, English Springer Spaniel, Manchester Terrier, Toy Poodle, Airedale Terrier, Bearded Collie, Belgian Sheepdog, Dutch Sheepdog, English Setter, Great Pyrenees, Irish Wolfhound, Miniature Dachshund, Shiba, Shiloh Shepherd, Terrier | 1,283 (10.9) |
| Exotic mammal | Mink, Porcupine, Fox, Deer, Hedgehog, Gray Seal, Rat, Ferret, Rabbit, Mouse, Alpaca, Lion, Squirrel, Guinea Pig, Llama, Raccoon, Harbour Porpoise, Tiger, Beaver, Moose, Otter, Hamster, Harbour Seal, Lynx, Monkey, Water Buffalo, Chinchilla, Zebra, White-beaked Dolphine | 1,024 (8.6) |
| Avian | Chicken, Turkey, Crow, Pigeon, Cockatiel, Finch, Moluccan Cockatoo, Mixed, Parakeet, Conure, Great Horned Owl, African Gray Parrot, Duck, Mourning Dove, Falcon, Blue Fronted Amazon Parrot, Blue Jay, Macaw, Red-Tailed Hawk, American Kestrel, Hawk | 970 (8.2) |
| Ovine | Mixed, North County Cheviot, Dorset, Hampshire, Suffolk | 864 (7.3) |
| Feline | Domestic Shorthair, Domestic Longhair, Persian, Birman, Abyssinian, Bengal | 525 (4.5) |
| Exotic reptile | Snake, Lizard, Turtle, Chameleon, Boa Constrictor, Bearded Dragon, Ball python, gecko, | 389 (3.3) |
| Caprine | Angora, Alpine, Nubian, Saanen | 193 (1.6) |
| Exotic avian | Senegal Parrot | 4 (0.03) |
| **Total samples submitted** **that met the inclusion criteria** |  | **11,792 (100.0)** |

**Supplementary Table 2.** Frequency of anatomic sites and specimen types submitted that met the inclusion criteria (2012 – 2021)

| **Anatomic sites submitted** | **Frequency**  **N (%)** |
| --- | --- |
| Feces | 4,660 (39.5) |
| Intestine | 2,840 (24.1) |
| Others | 2,010 (17.0) |
| Lung | 1,131 (9.6) |
| Liver | 724 (6.1) |
| Gallbladder | 252 (2.1) |
| Kidney | 164 (1.4) |
| Unknown | 11 (0.1) |
| **Total** | **11,792 (100.0)** |
|  |  |
| **Specimens submitted** | **Frequency**  **N (%)** |
| Tissue | 5,245 (44.5) |
| Unknown | 3,399 (28.8) |
| Swab | 2,331 (19.8) |
| Fluid | 441 (3.7) |
| Feces | 367 (3.1) |
| Plate | 9 (0.1) |
| **Total** | **11,792 (100.0)** |

Others include abdominal cavity, abomasum, abscess, air sac, anal sac, aspirate, bile, bladder, blood, bone, brain, cecum, choana, cloaca, coelom, colon, crop, diverticulum, duodenum, ear, esophagus, eye, fetus, floor sample, granuloma, heart, ileum, jejunum, joint, lymph node, mammary tissue, mass, meninges, mouth, nasal, oropharynx, ovary, oviduct, pancreas, perianal, pericardial cavity, peritoneal cavity, placenta, pleural cavity, prepuce, prostate, rectum, scrotum, sinus, skin, spleen, stomach, subcutaneous, thorax, throat, trachea, umbilicus, urine, uterus, wound, and yolk sac

**Supplementary Table 3.** Frequency of *Salmonella* *enterica* serotypes isolated from samples of various animal species that met the inclusion criteria (2012 – 2021)

| **A.) Exotic mammals**   \| ***Salmonella* *enterica* serotype in exotic mammal** \| **Samples of exotic mammal breeds submitted** \| **Frequency of *salmonella* *enterica* serotype in exotic mammal’s breed sample**  **N (%)** \| \| --- \| --- \| --- \| \| Typhimurium  N = 57 \| Mouse \| 22 (38.6) \| \|  \| Hedgehog \| 19 (33.3) \| \|  \| Ferret \| 16 (28.1) \| \| Heidelberg  N = 51 \| Porcupine \| 19 (37.3) \| \|  \| Mink \| 16 (31.4) \| \|  \| Lion \| 16 (31.4) \| \| Kentucky  N = 17 \| Mink \| 17 (100.0) \| \| Berta  N = 16 \| Mink \| 16 (100.0) \| \| Dublin  N = 16 \| Mink \| 16 (100.0) \| \| I 4, [5],12:i:-  N = 1 \| Harbour Porpoise \| 1 (10.0) \| \|  \| \| I:4,12:-:-  N= 2 \| Harbour Porpoise \| 1 (50.0) \| \|  \| White-beaked Dolphin \| 1 (50.0) \| \| Typhimurium var*.* Copenhagen  N = 1 \| Porcupine \| 1 (100.0) \| \| Hadar  N = 1 \| Porcupine \| 1 (100.0) \| \| Untypable  N = 53 \| Deer \| 27 (50.9) \| \|  \| Porcupine \| 23 (43.4) \| \|  \| Lion \| 1 (1.9) \| \|  \| Mink \| 1 (1.9) \| \|  \| Harbour Porpoise \| 1 (1.9) \|   **B.)** **Exotic reptiles**   \| ***Salmonella* *enterica* serotype in exotic reptile** \| **Samples of exotic reptile breeds submitted** \| **Frequency of *salmonella* *enterica* serotype in exotic reptile’s breed sample**  **N (%)** \| \| --- \| --- \| --- \| \| Kisarawe  N = 32 \| Bearded Dragon \| 32 (100.0) \| \| Fluntern  N = 22 \| Lizard \| 22 (100.0) \| \| Muenster  N = 22 \| Lizard \| 22 (100.0) \| \| II:16:m,t:-  N = 22 \| Snake \| 21 (95.5) \| \|  \| Ball Python \| 1 (4.5) \| \| IV:16:-:-  N = 19 \| Snake \| 19 (100.0) \| \| IV (44) z4  N = 19 \| Bearded Dragon \| 18 (94.7) \| \|  \| Gecko \| 1 (5.3) \| \| Blijdorp  N = 16 \| Chameleon \| 16 (100.0) \| \| IIIb:48:z4,z24:-  N = 16 \| Snake \| 16 (100.0) \| \| Mountpleasant  N = 16 \| Lizard \| 16 (100.0) \| \| Paratyphi  N = 5 \| Snake \| 5 (100.0) \| \| Hadar  N = 4 \| Lizard \| 2 (50.0) \| \|  \| Bearded Dragon \| 2 (50.0) \| \| Blukwa  N = 2 \| Snake \| 2 (100.0) \| \| I:4, [5],12:i:-  N = 1 \| Lizard \| 1 (100.0) \| \| II:58:I,z13,z28:z6  N = 1 \| Lizard \| 1 (100.0) \| \| IIIb:48:k:e,n,x,z15  N = 1 \| Snake \| 1 (100.0) \| \| IIIb:53:z10:z35  N = 1 \| Snake \| 1 (100.0) \| \| IIIb:60:i:e,n,x,z15  N = 1 \| Turtle \| 1 (100.0) \| \| IIIb:rough-O:z10  N = 1 \| Bearded Dragon \| 1 (100.0) \| \| IV:rough-O:gz10  N = 1 \| Bearded Dragon \| 1 (100.0) \| \| IIIa:18:-:-  N = 1 \| Snake \| 1 (100.0) \| \| IV 50:g,z51:-  N = 1 \| Bearded Dragon \| 1 (100.0) \| \| Miami  N = 1 \| Boa Constrictor \| 1 (100.0) \| \| Poona  N = 1 \| Snake \| 1 (100.0) \| \| Bareilly  N = 1 \| Snake \| 1 (100.0) \| \| Benin  N = 1 \| Snake \| 1 (100.0) \| \| Cerro  N = 1 \| Snake \| 1 (100.0) \| \| Cotham  N = 1 \| Bearded Dragon \| 1 (100.0) \| \| Muenchen  N = 1 \| Snake \| 1 (100.0) \| \| Rosslyn  N = 1 \| Snake \| 1 (100.0) \| \| Untypable  N = 7 \| Snake \| 2 (28.6) \| \|  \| Turtle \| 2 (28.6) \| \|  \| Bearded Dragon \| 1 (14.3) \| \|  \| Lizard \| 1 (14.3) \| \|  \| Gecko \| 1 (14.3) \|   **C.) Bovine**   \| ***Salmonella* *enterica* serotype in bovine** \| **Samples of bovine breeds submitted** \| **Frequency of *Salmonella enterica* serotype in bovine breed sample**  **N (%)** \| \| --- \| --- \| --- \| \| Enteritidis  N = 17 \| Holstein Friesian \| 1 (5.9) \| \|  \| Unknown \| 16 (94.1) \| \| Mbandaka  N = 16 \| Unknown \| 16 (100.0) \| \| Heidelberg  N = 8 \| Holstein Friesian \| 8 (100.0) \| \| *Salmonella diarizonae*  **N =** 1 \| Unknown \| 1 (100.0) \| \| Untypable  N = 30 \| Holstein Friesian \| 16 (53.3) \| \|  \| Unknown \| 14 (46.7) \|   **D.) Porcine**   \| ***Salmonella* *enterica* serotype in porcine** \| **Samples of porcine breed submitted.** \| **Frequency of *Salmonella* *enterica* serotype in porcine breed sample**  **N (%)** \| \| --- \| --- \| --- \| \| Typhimurium  N = 79 \| Mixed \| 17 (21.5) \| \|  \| Unknown \| 62 (78.5) \| \| Derby  N = 75 \| Unknown \| 75 (100.0) \| \| Typhimurium var*.* Copenhagen  N = 57 \| Unknown \| 57 (100.0) \| \| Infantis  N = 37 \| Mixed \| 20 (54.1) \| \|  \| Unknown \| 17 (45.9) \| \| Mbandaka  N = 29 \| Unknown \| 29 (100.0) \| \| I:4, [5],12:i:-  N = 17 \| Mixed \| 17 (100.0) \| \| Uganda  N = 9 \| Unknown \| 9 (100.0) \| \| Orion  N = 9 \| Unknown \| 9 (100.0) \| \| Untypable  N = 80 \| Mixed \| 9 (11.3) \| \|  \| Unknown \| 71 (88.8) \|   **E.) Ovine**   \| ***Salmonella* *enterica* serotype in ovine** \| **Breed** \| **Frequency of *Salmonella* *enterica* serotype in ovine breed sample**  **N (%)** \| \| --- \| --- \| --- \| \| IIIb:61:k:1,5  N = 77 \| Mixed \| 50 (65.8) \| \|  \| Unknown \| 27 (35.5) \| \| Typhimurium  N = 2 \| Unknown \| 2 (100.0) \| \| Untypable  N = 2 \| Unknown \| 2 (100.0) \|   **F.) Equine**   \| ***Salmonella* *enterica* serotype in equine** \| **Samples of equine breeds submitted** \| **Frequency of *Salmonella* *enterica* serotype in equine breed sample**  **N (%)** \| \| --- \| --- \| --- \| \| Anatum  N = 21 \| Miniature \| 21 (100.0) \| \| Typhimurium var*.* Copenhagen  N = 20 \| Standardbred \| 20 (100.0) \| \| Oranienburg  N = 16 \| Quarter \| 16 (100.0) \| \| Infantis  N = 9 \| Standardbred \| 9 (100.0) \| \| Kentucky  N = 8 \| Hanoverian \| 8 (100.0) \| \| Senftenberg  N = 1 \| Quarter \| 1 (100.0) \| \| Barranquilla  N = 1 \| Warmblood \| 1 (100.0) \| \| Untypable  N = 38 \| Standardbred \| 20 (52.6) \| \|  \| Warmblood \| 18 (47.4) \|   **G.) Canine**   \| ***Salmonella* *enterica* serotype in canine** \| **Samples of canine breeds submitted** \| **Frequency of *Salmonella* *enterica* serotype in canine breed sample**  **N (%)** \| \| --- \| --- \| --- \| \| Enteritidis  N = 38 \| French Bulldog \| 19 (50.0) \| \|  \| Golden Retriever \| 19 (50.0) \| \| Heidelberg  N = 32 \| Great Dane \| 16 (50.0) \| \|  \| Mixed \| 16 (50.0) \| \| Panama  N = 22 \| Rottweiler \| 22 (100.0) \| \| I:rough-O:d:l:,w  N = 19 \| Pug \| 19 (100.0) \| \| Reading  N = 19 \| Staffordshire Terrier \| 19 (100.0) \| \| Tennessee  N = 19 \| Mixed \| 19 (100.0) \| \| Hadar  N = 19 \| Golden Retriever \| 19 (100.0) \| \| I:4, [5],12:i:-  N = 17 \| Mixed \| 16 (94.1) \| \|  \| Doberman Pinscher \| 1 (5.9) \| \| Typhimurium  N = 16 \| Mixed \| 16 (100.0) \| \| Typhimurium var*.* Copenhagen  N = 16 \| Unknown \| 16 (100.0) \| \| I:rough-O:d:l:,w  N = 7 \| Poodle \| 100 (100.0) \| \| Heidelberg  N = 7 \| Bull Mastiff \| 7 (100.0) \| \| *Salmonella diarizonae*  N = 1 \| Labrador Retriever \| 1 (100.0) \| \| Newport  N = 1 \| Mixed \| 1 (100.0) \| \| Untypable  N = 75 \| Poodle \| 33 (44.0) \| \|  \| Great Dane \| 19 (25.3) \| \|  \| Unknown \| 19 (25.3) \| \|  \| Mixed \| 3 (4.0) \| \|  \| French Bulldog \| 1 (1.3) \| |  |  |  |  |  |  |
| --- | --- | --- | --- | --- | --- | --- | --- | --- | --- | --- | --- | --- | --- | --- | --- | --- | --- | --- | --- | --- | --- | --- | --- | --- | --- | --- | --- | --- | --- | --- | --- | --- | --- | --- | --- | --- | --- | --- | --- | --- | --- | --- | --- | --- | --- | --- | --- | --- | --- | --- | --- | --- | --- | --- | --- | --- | --- | --- | --- | --- | --- | --- | --- | --- | --- | --- | --- | --- | --- | --- | --- | --- | --- | --- | --- | --- | --- | --- | --- | --- | --- | --- | --- | --- | --- | --- | --- | --- | --- | --- | --- | --- | --- | --- | --- | --- | --- | --- | --- | --- | --- | --- | --- | --- | --- | --- | --- | --- | --- | --- | --- | --- | --- | --- | --- | --- | --- | --- | --- | --- | --- | --- | --- | --- | --- | --- | --- | --- | --- | --- | --- | --- | --- | --- | --- | --- | --- | --- | --- | --- | --- | --- | --- | --- | --- | --- | --- | --- | --- | --- | --- | --- | --- | --- | --- | --- | --- | --- | --- | --- | --- | --- | --- | --- | --- | --- | --- | --- | --- | --- | --- | --- | --- | --- | --- | --- | --- | --- | --- | --- | --- | --- | --- | --- | --- | --- | --- | --- | --- | --- | --- | --- | --- | --- | --- | --- | --- | --- | --- | --- | --- | --- | --- | --- | --- | --- | --- | --- | --- | --- | --- | --- | --- | --- | --- | --- | --- | --- | --- | --- | --- | --- | --- | --- | --- | --- | --- | --- | --- | --- | --- | --- | --- | --- | --- | --- | --- | --- | --- | --- | --- | --- | --- | --- | --- | --- | --- | --- | --- | --- | --- | --- | --- | --- | --- | --- | --- | --- | --- | --- | --- | --- | --- | --- | --- | --- | --- | --- | --- | --- | --- | --- | --- | --- | --- | --- | --- | --- | --- | --- | --- | --- | --- | --- | --- | --- | --- | --- | --- | --- | --- | --- | --- | --- | --- | --- | --- | --- | --- | --- | --- | --- | --- | --- | --- | --- | --- | --- | --- | --- | --- | --- | --- | --- | --- | --- | --- | --- | --- | --- | --- | --- | --- | --- | --- | --- | --- | --- | --- | --- | --- | --- | --- | --- | --- | --- | --- | --- | --- | --- | --- | --- | --- | --- | --- | --- | --- | --- | --- | --- | --- | --- | --- | --- | --- | --- | --- | --- |

**H.) Feline**

| ***Salmonella* *enterica* serotype in feline** | **Samples of feline breeds submitted** | **Frequency of *Salmonella enterica* serotype in feline breed sample**  **N (%)** |
| --- | --- | --- |
| IIIb:rough-O:z10:e,n,x,z15  N = 19 | Persian | 19 (100.0) |
| Heidelberg  N = 16 | Unknown | 16 (100.0) |
| Untypable  N = 1 | Persian | 1 (100.0) |

**I.) Avian**

| ***Salmonella* *enterica* serotype in avian** | **Samples of avian breeds submitted** | **Frequency of *Salmonella* *enterica* serotype in avian breed sample**  **N (%)** |
| --- | --- | --- |
| Muenchen  N = 24 | Turkey | 24 (100.0) |
| Typhimurium var*.* Copenhagen  N = 16 | Pigeon | 16 (100.0) |
| Bredeney  N = 9 | Turkey | 9 (100.0) |
| I:4, [5],12:i:-  N = 3 | Unknown | 2 (66.7) |
|  | Great Horned Owl | 1 (33.3) |
| Typhimurium  N = 1 | Gull | 1 (100.0) |
| Senftenberg  N = 1 | Turkey | 1 (100.0) |
| Untypable  N = 12 | Unknown | 8 (66.7) |
|  | Finch | 3 (25.0) |
|  | Turkey | 1 (8.3) |

**Supplementary Table 4:** Antimicrobials tested (and their disk content) and the applied CLSI susceptibility breakpoints (in µg/mL) for *Salmonella* *enterica* isolates from various samples of animal species (2012 – 2021). NT = Not tested.

| **Antimicrobial**  **(disk content)** | **Avian** | **Bovine** | **Canine** | **Caprine** | **Equine** | **Exotic mammal** | **Exotic reptiles** | **Feline** | **Ovine** | **Porcine** |
| --- | --- | --- | --- | --- | --- | --- | --- | --- | --- | --- |
| Ampicillin  (10 ug) | ≤ 8 (poultry)  ≤ 0.25 (exotic) | ≤ 8 | ≤ 0.25 | ≤ 8 | ≤ 0.25 | ≤ 0.25 | ≤ 0.25 | ≤ 0.25 | ≤ 8 | ≤ 8 |
| amoxicillin-clavulanate (20/10 ug) | NT | NT | ≤ 0.25 | NT | NT | ≤ 0.25 | ≤ 0.25 | ≤ 0.25 | NT | NT |
| Ceftiofur  (30 ug) | ≤ 2 | ≤ 2 | NT | ≤ 2 | ≤ 2 | ≤ 2 | ≤ 2 | NT | ≤ 2 | ≤ 2 |
| Cefovecin  (30 ug) | NT | NT | ≤ 2 | NT | NT | ≤ 2 | ≤ 2 | ≤ 2 | NT | NT |
| Cefpodoxime  (10 ug) | NT | NT | ≤ 2 | NT | NT | ≤ 2 | ≤ 2 | ≤ 2 | NT | NT |
| Chloramphenicol (30 ug) | NT | NT | ≤ 8 | NT | ≤ 8 | ≤ 8 | ≤ 8 | ≤ 8 | NT | NT |
| Doxycycline  (30 ug) | NT | NT | ≤ 4 | NT | ≤ 4  (2015-2018)  ≤ 0.12  (after 2018) | ≤ 4 | ≤ 4 | ≤ 4 | NT | NT |
| Enrofloxacin  (5 ug) | ≤ 0.25 | ≤ 0.5 | ≤ 0.5 | ≤ 0.5 | ≤ 0.5  (2015-2018)  ≤ 0.12  (after 2018) | ≤ 0.5 | ≤ 0.5 | ≤ 0.5 | ≤ 0.5 | ≤ 0. 5 |
| Florfenicol  (30 ug) | ≤ 4 | ≤ 4 | NT | ≤ 4 | NT | NT | NT | NT | ≤ 4 | ≤ 4 |
| Marbofloxacin  (5 ug) | NT | NT | ≤ 1 | NT | NT | ≤ 1 | NT | ≤ 1 | NT | NT |
| Oxytetracycline (30 ug) | ≤ 4 | ≤ 4 | NT | ≤ 4 | NT | NT | NT | NT | ≤ 4 | ≤ 4 |
| Sulfadimethoxine (Not used) | ≤ 256 | ≤ 256 | NT | ≤ 256 | NT | ≤ 256 | NT | NT | ≤ 256 | ≤ 256 |
| trimethoprim-sulfamethoxazole (1.25/23.75 ug) | ≤ 2 | ≤ 2 | ≤ 2 | ≤ 2 | ≤ 2 | ≤ 2 | ≤ 2 | ≤ 2 | ≤ 2 | ≤ 2 |
| Tiamulin  (30 ug) | + | + | NT | + | NT | + | NT | NT | + | + |

**Supplementary Table 5.** Antimicrobial susceptibility profiles (% S and NS) for tested *Salmonella* serotypes isolated from animal species samples (2012 – 2021)

|  |  | | | | | | | | | | | | | | | |  |  |  |
| --- | --- | --- | --- | --- | --- | --- | --- | --- | --- | --- | --- | --- | --- | --- | --- | --- | --- | --- | --- |
|  |  |  | **Ampicillin** | |  | **Ceftiofur** | |  | **Cefovecin** | |  | **Chloramphenicol** | |  | **Doxycycline** | |  | **Enrofloxacin** | |
| **Animal species** | **Bacterial isolates** | **N** | **S** | **NS** | **N** | **S** | **NS** | **N** | **S** | **NS** | **N** | **S** | **NS** | **N** | **S** | **NS** | **N** | **S** | **NS** |
| Exotic mammals | Typhimurium | 3 | 33.3 | 66.7 | 7 | 100.0 | 0.0 | 2 | 50.0 | 50.0 | 3 | 100.0 | 0.0 | 2 | 100.0 | 0.0 | - | - | - |
|  | Derby | - | - | - | 7 | 100.0 | 0.0 | - | - | - | - | - | - | - | - | - | - | - | - |
|  | Typhimurium var*.* Copenhagen | - | - | - | 5 | 100.0 | 0.0 | - | - | - | - | - | - | - | - | - | - | - | - |
|  | Mbandaka | - | - | - | 3 | 100.0 | 0.0 | - | - | - | - | - | - | - | - | - | - | - | - |
|  | Infantis | - | - | - | 2 | 100.0 | 0.0 | - | - | - | - | - | - | - | - | - | - | - | - |
|  | Heidelberg | 3 | 100.0 | 0.0 | - | - | - | - | - | - | 3 | 100.0 | 0.0 | - | - | - | - | - | - |
| Mink | Dublin | 1 | 100.0 | 0.0 | - | - | - | 1 | 0.0 | 100.0 | 1 | 0.0 | 100.0 | 1 | 0.0 | 100.0 | 1 | 100.0 | 0.0 |
|  | Kentucky | 1 | 0.0 | 100.0 | - | - | - | 1 | 0.0 | 100.0 | 1 | 100.0 | 0.0 | 1 | 0.0 | 100.0 | 1 | 100.0 | 0.0 |
|  | Berta | 1 | 100.0 | 0.0 | - | - | - | 1 | 100.0 | 0.0 | 1 | 100.0 | 0.0 | 1 | 100.0 | 0.0 | 1 | 100.0 | 0.0 |
| Exotic reptiles | IIIb:48:k:e,n,x,z15 | 2 | 100.0 | 0.0 | - | - | - | - | - | - | 2 | 100.0 | 0.0 | 2 | 100.0 | 0.0 | 2 | 100.0 | 0.0 |
| Canine | Enteritidis | 2 | 0.0 | 100.0 | - | - | - | 2 | 100.0 | 0.0 | 2 | 100.0 | 0.0 | 2 | 50.0 | 50.0 | 2 | 100.0 | 0.0 |
|  | Heidelberg | 2 | 100.0 | 0.0 | - | - | - | - | - | - | 2 | 100.0 | 0.0 | 2 | 100.0 | 0.0 | 2 | 100.0 | 0.0 |
| Avian | Muenchen | - | - | - | 2 | 100.0 | 0.0 | - | - | - | - | - | - | - | - | - | - | - | - |
| Ovine | IIIb:61:k:1,5 | 4 | 100.0 | 0.0 | 5 | 100.0 | 0.0 | 5 | 100.0 | 0.0 | - | - | - | - | - | - | 4 | 100.0 | 0.0 |
|  | Typhimurium | - | - | - | 7 | 100.0 | 0.0 | - | - | - | - | - | - | - | - | - | 2 | 100.0 | 0.0 |
|  | Derby | - | - | - | 7 | 100.0 | 0.0 | - | - | - | - | - | - | - | - | - | - | - | - |
| Porcine | Infantis | - | - | - | 3 | 75.0 | 25.0 | - | - | - | - | - | - | - | - | - | - | - | - |
|  | Typhimurium var*.* Copenhagen | - | - | - | 5 | 100.0 | 0.0 | - | - | - | - | - | - | - | - | - | - | - | - |
|  | Mbandaka | - | - | - | 3 | 100.0 | 0.0 | - | - | - | - | - | - | - | - | - | - | - | - |
|  |  |  |  |  |  |  |  |  |  |  |  |  |  |  |  |  |  |  |  |

**N =** number of isolates tested, **S** - % Susceptible, **NS** - % Not susceptible

|  |  |  |  |  |  |  |  |  |  |  |  |  |  |
| --- | --- | --- | --- | --- | --- | --- | --- | --- | --- | --- | --- | --- | --- |
|  |  |  | **Oxytetracycline** | |  | **Sulfadimethoxine** | |  | **Trimethoprim-sulfamethoxazole** | |  | **Marbofloxacin** | |
| **Animal species** | **Bacterial isolates** | **N** | **S** | **NS** | **N** | **S** | **NS** | **N** | **S** | **NS** | **N** | **S** | **NS** |
| Exotic mammals | Typhimurium | 6 | 0.0 | 100.0 | 2 | 0.0 | 100.0 | 7 | 100.0 | 0.0 | - | - | - |
|  | Derby | 7 | 100.0 | 0.0 | - | - | - | 7 | 100.0 | 0.0 | - | - | - |
|  | Typhimurium var*.* Copenhagen | 5 | 0.0 | 100.0 | - | - | - | 5 | 100.0 | 0.0 | - | - | - |
|  | Mbandaka | 3 | 100.0 | 0.0 | - | - | - | 3 | 100.0 | 0.0 | - | - | - |
|  | Infantis | 3 | 100.0 | 0.0 | - | - | - | 3 | 100.0 | 0.0 | - | - | - |
|  | Heidelberg | - | - | - | - | - | - | - | - | - | - | - | - |
| Mink | Dublin | - | - | - | - | - | - | 1 | 100.0 | 0.0 | 1 | 100.0 | 0.0 |
|  | Kentucky | - | - | - | - | - | - | 1 | 100.0 | 0.0 | 1 | 100.0 | 0.0 |
|  | Berta | - | - | - | - | - | - | 1 | 100.0 | 0.0 | 1 | 100.0 | 0.0 |
| Canine | Enteritidis | - | - | - | - | - | - | 2 | 100.0 | 0.0 | 2 | 100.0 | 0.0 |
|  | Heidelberg | - | - | - | - | - | - | 2 | 100.0 | 0.0 | - | - | - |
| Avian | Muenchen | 2 | 50.0 | 50.0 | - | - | - | 2 | 100.0 | 0.0 | - | - | - |
| Ovine | IIIb:61:k:1,5 | 3 | 100.0 | 0.0 | 4 | 50.0 | 50.0 | 5 | 100.0 | 0.0 | - | - | - |
|  | Typhimurium | 6 | 0.0 | 100.0 | 2 | 0.0 | 100.0 | 7 | 100.0 | 0.0 | - | - | - |
|  | Derby | 7 | 100.0 | 0.0 | - | - | - | 7 | 100.0 | 0.0 | - | - | - |
| Porcine | Infantis | 3 | 100.0 | 0.0 | - | - | - | 3 | 100.0 | 0.0 | - | - | - |
|  | Typhimurium var*.* Copenhagen | 5 | 100.0 | 0.0 | - | - | - | 5 | 100.0 | 0.0 | - | - | - |
|  | Mbandaka | 3 | 100.0 | 0.0 | - | - | - | 3 | 100.0 | 0.0 | - | - | - |
|  |  |  |  |  |  |  |  |  |  |  |  |  |  |

**N =** number of isolates tested, **S** - % Susceptible, **NS** - % Not susceptible

**Supplementary Table 6A (metadata):** Annual and monthly frequency of cases submitted to AVC DSBL for bacterial culture and identification that met the inclusion criteria (2012 – 2021)

|  |  | | | | | | | | | | |
| --- | --- | --- | --- | --- | --- | --- | --- | --- | --- | --- | --- |
| **Month** | **2012** | **2013** | **2014** | **2015** | **2016** | **2017** | **2018** | **2019** | **2020** | **2021** | **Average no. of cases** |
| Jan | 30 | 33 | 32 | 28 | 29 | 14 | 18 | 13 | 12 | 21 | 23.0 |
| Feb | 30 | 33 | 21 | 24 | 34 | 9 | 12 | 14 | 12 | 12 | 20.1 |
| Mar | 30 | 37 | 44 | 36 | 33 | 20 | 25 | 8 | 11 | 18 | 26.2 |
| Apr | 35 | 33 | 28 | 43 | 39 | 16 | 15 | 10 | 7 | 15 | 24.1 |
| May | 56 | 48 | 41 | 50 | 22 | 52 | 24 | 29 | 12 | 23 | 33.4 |
| Jun | 37 | 44 | 39 | 46 | 9 | 26 | 19 | 17 | 9 | 40 | 28.6 |
| Jul | 38 | 43 | 32 | 32 | 6 | 17 | 14 | 25 | 29 | 39 | 27.5 |
| Aug | 41 | 47 | 29 | 34 | 9 | 12 | 18 | 6 | 11 | 39 | 24.6 |
| Sep | 55 | 51 | 59 | 54 | 19 | 41 | 35 | 61 | 15 | 22 | 41.2 |
| Oct | 53 | 50 | 44 | 39 | 26 | 28 | 42 | 31 | 26 | 36 | 37.5 |
| Nov | 29 | 25 | 34 | 43 | 10 | 26 | 18 | 18 | 10 | 28 | 24.1 |
| Dec | 27 | 17 | 56 | 19 | 7 | 15 | 11 | 13 | 21 | 30 | 21.6 |
| **Total** | **461** | **461** | **459** | **448** | **243** | **276** | **251** | **245** | **175** | **323** |  |
|  |  |  |  |  |  |  |  |  |  |  |  |
|  |  |  |  |  |  |  |  |  |  |  |  |
|  |  |  |  |  |  |  |  |  |  |  |  |

**Supplementary Table 6B (metadata):** Annual trends in antimicrobial resistance for *Salmonella* *enterica* serotypes most frequently tested in porcine samples (2012 - 2021).

| **Antimicrobial** | ***Salmonella* serotype** | **Year** | **No. of isolates** | **Susceptible** | **Intermediate** | **Resistant** |
| --- | --- | --- | --- | --- | --- | --- |
| Ampicillin | Typhimurium | 2012 | 18 | 0.0 | 0.0 | 100.0 |
|  |  | 2015 | 18 | 0.0 | 0.0 | 100.0 |
|  |  | 2017 | 1 | 0.0 | 0.0 | 100.0 |
|  |  | 2020 | 1 | 100.0 | 0.0 | 0.0 |
|  | Derby | 2013 | 9 | 100.0 | 0.0 | 0.0 |
|  |  | 2014 | 27 | 0.0 | 0.0 | 100.0 |
|  |  | 2019 | 1 | 100.0 | 0.0 | 0.0 |
|  | Typhimurium var. Copenhagen | 2013 | 27 | 0.0 | 0.0 | 100.0 |
|  |  | 2014 | 9 | 0.0 | 0.0 | 100.0 |
|  |  | 2017 | 1 | 0.0 | 0.0 | 100.0 |
|  | Mbandaka | 2012 | 27 | 100.0 | 0.0 | 0.0 |
|  |  | 2013 | 1 | 100.0 | 0.0 | 0.0 |
|  | Infantis | 2014 | 9 | 100.0 | 0.0 | 0.0 |
|  |  | 2015 | 10 | 10.0 | 0.0 | 90.0 |
|  | Uganda | 2012 | 9 | 100.0 | 0.0 | 0.0 |
|  | Orion | 2013 | 9 | 100.0 | 0.0 | 0.0 |
|  |  |  |  |  |  |  |
| Florfenicol | Typhimurium | 2015 | 18 | 50.0 | 0.0 | 50.0 |
|  |  | 2017 | 16 | 100.0 | 0.0 | 0.0 |
|  |  | 2020 | 1 | 100.0 | 0.0 | 0.0 |
|  | Derby | 2013 | 9 | 100.0 | 0.0 | 0.0 |
|  |  | 2014 | 27 | 100.0 | 0.0 | 0.0 |
|  |  | 2019 | 16 | 100.0 | 0.0 | 0.0 |
|  | Typhimurium var. Copenhagen | 2013 | 27 | 0.0 | 0.0 | 100.0 |
|  |  | 2014 | 9 | 0.0 | 0.0 | 100.0 |
|  | I:4,[5],12:i:- | 2017 | 16 | 0.0 | 0.0 | 100.0 |
|  | Infantis | 2014 | 9 | 100.0 | 0.0 | 0.0 |
|  |  | 2015 | 9 | 100.0 | 0.0 | 0.0 |
|  | Orion | 2013 | 9 | 100.0 | 0.0 | 0.0 |
|  |  |  |  |  |  |  |
